# Application of anti-fungal vaccines as a tool against emerging anti-fungal resistance

**DOI:** 10.3389/ffunb.2023.1241539

**Published:** 2023-08-21

**Authors:** Ravinder Kumar, Vartika Srivastava

**Affiliations:** ^1^ Department of Pathology, Collage of Medicine, University of Tennessee Health Science Center, Memphis, TN, United States; ^2^ Department of Clinical Microbiology and Infectious Diseases, School of Pathology, Faculty of Health Sciences, University of the Witwatersrand, Johannesburg, Gauteng, South Africa

**Keywords:** anti-fungal, resistance, vaccines, infection, mRNA, endemic

## Abstract

After viruses and bacteria, fungal infections remain a serious threat to the survival and well-being of society. The continuous emergence of resistance against commonly used anti-fungal drugs is a serious concern. The eukaryotic nature of fungal cells makes the identification of novel anti-fungal agents slow and difficult. Increasing global temperature and a humid environment conducive to fungal growth may lead to a fungal endemic or a pandemic. The continuous increase in the population of immunocompromised individuals and falling immunity forced pharmaceutical companies to look for alternative strategies for better managing the global fungal burden. Prevention of infectious diseases by vaccines can be the right choice. Recent success and safe application of mRNA-based vaccines can play a crucial role in our quest to overcome anti-fungal resistance. Expressing fungal cell surface proteins in human subjects using mRNA technology may be sufficient to raise immune response to protect against future fungal infection. The success of mRNA-based anti-fungal vaccines will heavily depend on the identification of fungal surface proteins which are highly immunogenic and have no or least side effects in human subjects. The present review discusses why it is essential to look for anti-fungal vaccines and how vaccines, in general, and mRNA-based vaccines, in particular, can be the right choice in tackling the problem of rising anti-fungal resistance.

## 1 Introduction

Fungus (pl: fungi) are a diverse group of organisms with the common characteristic of lacking chloroplast and the presence of a chitin cell wall (Naranjo-Ortiz and Gabaldón, 2019). Fungi belong to the eukaryotic domain of life (Woese et al., 1990). As predicted, around 2.2 to 6 million species of fungi may exist, although only around 120 000 have been described (Taylor et al., 2014; Hawksworth and Lücking, 2017). Fungi are natural decomposers essential in recycling environmental nutrients (Frąc et al., 2018). Apart from their vital role in the environment, fungi are also helpful to humans in several ways. For example, several species of fungi (known as a mushroom) are a source of food and nutrition (Ho et al., 2020). Fungi are essential in baking, beverage industries (beer, wine, alcohol), soya sauce, and cheese preparation. Fungi also found their importance in pharmaceutical companies. For example, drugs like ergometrine, cortisone, and cyclosporine are all derived from fungi. They also remain the source of essential antibiotics (for example, penicillin); also used for producing beneficial chemicals (like citric acid), a source of valuable enzymes, a host for the production of heterologous proteins, a biological model, and controlling pests in agriculture (Hyde et al., 2019).

Apart from their importance and benefits to human society (mentioned above), fungi have been the reason for great human suffering and economic loss on several occasions. For example, several species of fungi are pathogenic to farm animals such as cattle, poultry, fishery, and bees (Sexton and Howlett, 2006). Fungal infections are the most common and may have the potential for significant agricultural loss and human suffering. Moreover, most plant diseases are fungal. For example, the Irish potato famine (from 1845 -1852) caused by *Phytophthora infestans* led to widespread hunger and starvation in Europe, leading to 1 million deaths (Cantwell, 2017). Furthermore, fungi are also involved in the spoilage of food and other products (Ribes et al., 2018). Many fungi secrete toxic chemicals and infest food with fungal toxins, thus making it unsuitable for human consumption (Adeyeye, 2016). On the extreme side, fungi can potentially wipe out entire species. For example, *Batrachochytrium dendrobatidis* and *Batrachochytrium salamandrivorans* are responsible for the rapid decline of several amphibian species (Fisher and Garner, 2020).

Like viruses and bacteria, fungi are also known to cause several infectious diseases in animals, including humans. A disease or infection caused by fungi is known as mycosis (Richardson et al., 2012). Based on site, fungal infection can be superficial, cutaneous, subcutaneous, and systemic (affecting the entire body) (Dixon and Walsh, 1996). Although fungi are ubiquitous and out of millions of species, only around 600 fungal species are known to cause infections in humans (Richardson et al., 2012). Because of the reasons mentioned in the coming sections, treating, or managing fungal infections is becoming more challenging and a matter of great concern.

A healthy and immunocompetent individual maintaining optimum hygiene rarely gets a fungal infection. However, due to the rapid emergence and spread of anti-fungal resistance against commonly used anti-fungal drugs, there is a dire need to find alternative ways to treat better and manage the global fungal burden (Fisher et al., 2022). Discussion of the reasons and molecular mechanism involved in development of anti-fungal resistance is skipped in this review as this topic have been discussed by others (Cowen et al., 2015; Lee et al., 2021). This review will briefly discuss ways to better manage the increasing global fungal burden and rising anti-fungal resistance. We will focus on anti-fungal vaccines and will discuss how vaccines will be helpful in the better management of anti-fungal resistance. We will also highlight how mRNA-based vaccines can be a game changer. Before discussing anti-fungal vaccines, we will brief the readers about the global fungal burden, the economic impact of fungal infection in humans, and why it is vital to have anti-fungal vaccines. We will also discuss critical points that must be considered while choosing immunogens for anti-fungal vaccines and the different platforms used to develop anti-fungal vaccines. Different strategies used in the treatment of fungal infections are also discussed briefly. Factors contributing to the rise in global fungal burden are also highlighted.

## 2 The myth: fungal infections are rare

As mentioned above, a healthy individual rarely gets a fungal infection, but this does not mean that fungal infections are rare. In reality, fungal infections are also quite common; unfortunately, they either go unnoticed or undiagnosed most of the time. On most occasions, fungal infections are not reported. The fungal infection becomes apparent only when people visit doctors or when the condition becomes problematic and hard to treat. Further, there is no national policy to monitor fungal infections or diseases like viral or bacterial infections (CDC, ). Surprisingly even the WHO does not have any programme for global monitoring or surveillance of fungal diseases. Therefore, it is difficult to correctly estimate the actual number of fungal infections or fungal burden in the human population (Vallabhaneni et al., 2016).

However, several attempts were made to get an approximate estimate of the global fungal burden. According to one assessment worldwide, around 1.7 billion people suffer from superficial fungal infections (those of skin and hair) (Havlickova et al., 2008). Besides superficial infections, mucosal infections (genital tracts and mouth) are common. It is estimated that around 75 million women suffer from vulvovaginitis (Sobel, 2007). Interestingly, only four fungal genera, including *Aspergillus*, *Candida*, *Pneumocystis*, and *Cryptococcus*, are responsible for 90% (around 1.5 million) of all fungal-related deaths worldwide (Brown et al., 2012). Every year fungal infections kill approximately 1.3 million HIV-infected individuals worldwide, and this number is similar to the deaths caused by *Mycobacterium tuberculosis* and more than those from malaria (Brown et al., 2012). Again, it is essential to note that the actual number of total fungal infections and associated deaths from all fungal infections may be even more than that mentioned above.

Without national or global monitoring or surveillance of fungal burden, it is difficult to estimate the economic cost of fungal infection in humans precisely (Note, here we are not considering fungal infection in crop plants or farm animals). Fortunately, data collected by CDC in the USA can help estimate the economic cost of fungal infection in the American population. As per one estimate in the USA alone, the direct medical cost associated with fungal infections ranges from 6.7 to 7.5 billion USD annually (Benedict et al., 2019). Apart from the direct cost associated with a fungal infection, there is a significant amount of money due to indirect costs. The estimated indirect cost of fungal infection is around 4 billion USD in the USA alone (Benedict et al., 2022). According to another study, the total cost associated with a fungal infection may range from 11.5 to 48 billion USD annually in the USA (Benedict et al., 2022). Similarly, another independent study estimated the total cost associated with fungal infection (more than 666 000 diagnosed cases) in the USA in 2018 was around 37 billion USD, about 1.1% of the total US GDP (Medicalxpress). Again, as mentioned above, most fungal infection goes unnoticed; otherwise, the total economic burden due to fungal infection may be more than what is mentioned.

40-50 billion USD in the economics of more than 20 trillion USD may sound insignificant. Looking at this from another perspective, the total economic burden due to fungal infection in the USA every year is more than the entire GDP of several countries in Asia and Africa (Indicators, D. D). Although no precise estimate is available, it will not be surprising if the global economic burden due to fungal infection may run into several hundred billion USD. Apart from the financial burden, the suffering and loss of life caused by fungal infection globally are beyond economics. Therefore, it should be accepted that fungal infections are common and lead to substantial economic burdens, suffering, and loss of life. Therefore, fungal infections need attention equivalent to other microbial infections, including viral or bacterial.

## 3 Rising global fungal burden and possible fungal endemic or pandemic

**Figure 1:**
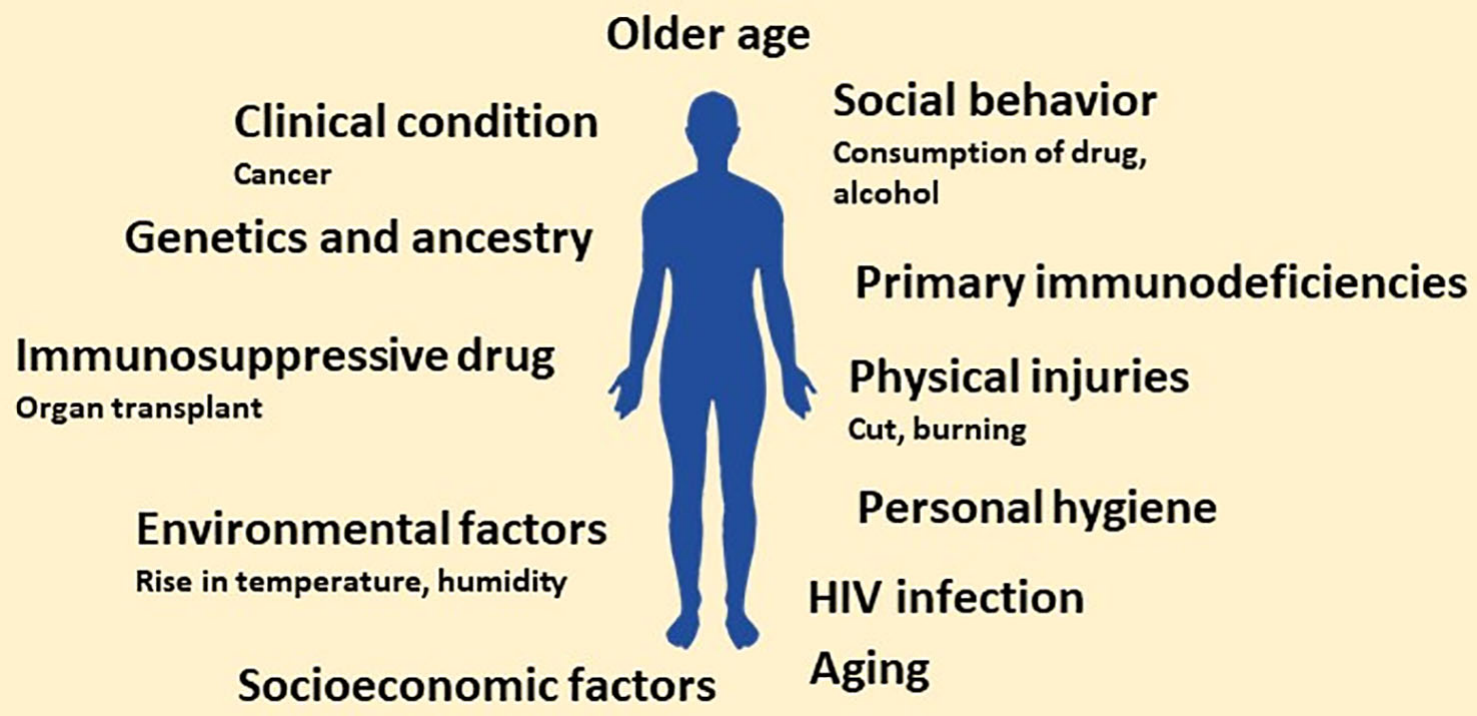
Schematic showing main risk factors predisposing to invasive fungal infections. In the future, the number of factors may increase owing to more research related to factors that increase the susceptibility to fungal infection.

Of millions of fungal species, only a few hundred are known to infect humans; unfortunately, despite their meagre number, the global fungal burden is continuously increasing (ENSIA, ). The increasing global fungal infection trend is also supported by the rising consumption of anti-fungal agents globally (Pathadka et al., 2022). The persistent rise in fungal infection or burden is attributed to several factors. The factors contributing to the persistent increase in global fungal burden may range from genetic to social and personal to the global environment. Discussion on each factor in detail is not possible in a single review. However, we will highlight a few studies showing each factor’s contribution to the increasing global fungal burden. Different factors contributing to the rising global fungal burden are shown in **Figure 1**.

The risk of getting a fungal infection is very high for immunocompromised individuals, and the scenario gets further complicated with the continuous increase in the number of these patients. Moreover, the rise in the population of immunocompromised individuals can be attributed to several factors. Firstly, the rapid increase in HIV patients (nearly 2 million newer cases of HIV infection every year) (CDC, ). Secondly, increased usage of immunosuppressive drugs due to organ transplants or surgery (Transplantation, ). Also, the number of cancer cases is rising yearly, resulting in patients with weak immunity (due to side effects of chemotherapy and radiation therapy) thereby, making them prone to fungal infections (Richardson, 2005; Wargo et al., 2015).

Additionally, there is a persistent increase in premature delivery or the population of neonates with poorly developed immune systems. These neonates without a well-developed immune system are at high risk of getting fungal infections, thus increasing the fungal burden in society (Shennan and Bewley, 2006; Hsieh et al., 2012; Vanderweele et al., 2012; Swanson et al., 2014). Further of more importance, individuals with primary immunodeficiencies (due to inherent genotype) also contribute to the global fungal burden (Antachopoulos et al., 2007; Lanternier et al., 2013; Lionakis, 2019; Meyts et al., 2021). It is well-accepted that an individual’s susceptibility to fungal infection may be linked to genetic endowment and ancestry (Naik et al., 2021). These immunocompromised individuals become an easy target of opportunistic pathogenic fungi like *Pneumocystis jiroveci* in HIV patients (Fujii et al., 2007), species of *Aspergillus*, *Candida and Cryptococcus* in patients with solid organ transplantation (Betancourt, 2019).

Apart from a compromised immune system, the individual’s social behaviour may also affect the number of fungal infections in the population; heavy alcohol consumption (Ali et al., 2020; Malacco et al., 2020), consumption of psychoactive substances or drugs (marijuana, heroin, LSD, cocaine, and amphetamines) (Friedman et al., 2006; Hadzic et al., 2013; Abharian et al., 2020) and smoking also increases the risk of fungal infection (Semlali et al., 2014; Pourbaix et al., 2020). Furthermore, socioeconomic factors, including poor hygiene due to a lack of basic facilities like access to clean drinking water, proper clean toilets, and the environment, may also predispose individuals to fungal infection (Olutoyin et al., 2017).

Another critical factor that increases the global fungal burden is the rise in global temperature and humidity (due to rainfall) favourable to fungal growth. In general, fungal cells prefer warm and humid climates for growth. The rise in global temperature and uncertain precipitation helps fungal growth even in areas or regions lacking obvious or significant fungal populations (Garcia-Solache and Casadevall, 2010; Nnadi and Carter, 2021; Gadre et al., 2022). The increasing number of aged people in the global population also contributes to the global fungal infection (Kauffman and Yoshikawa, 2001). Apart from old age, the increased pace of ageing also contributes to fungal infection (Piérard, 2001; Gong et al., 2020). Thus, looking at these, it can be said that several factors contribute to the increase in global fungal burden. Apart from the abovementioned attributes, several other factors must be identified contributing to the global fungal burden. Combining all these factors, the world may be heading toward a possible fungal pandemic.

## 4 Limited anti-fungal drugs and the emergence of anti-fungal resistance

**Figure 2:**
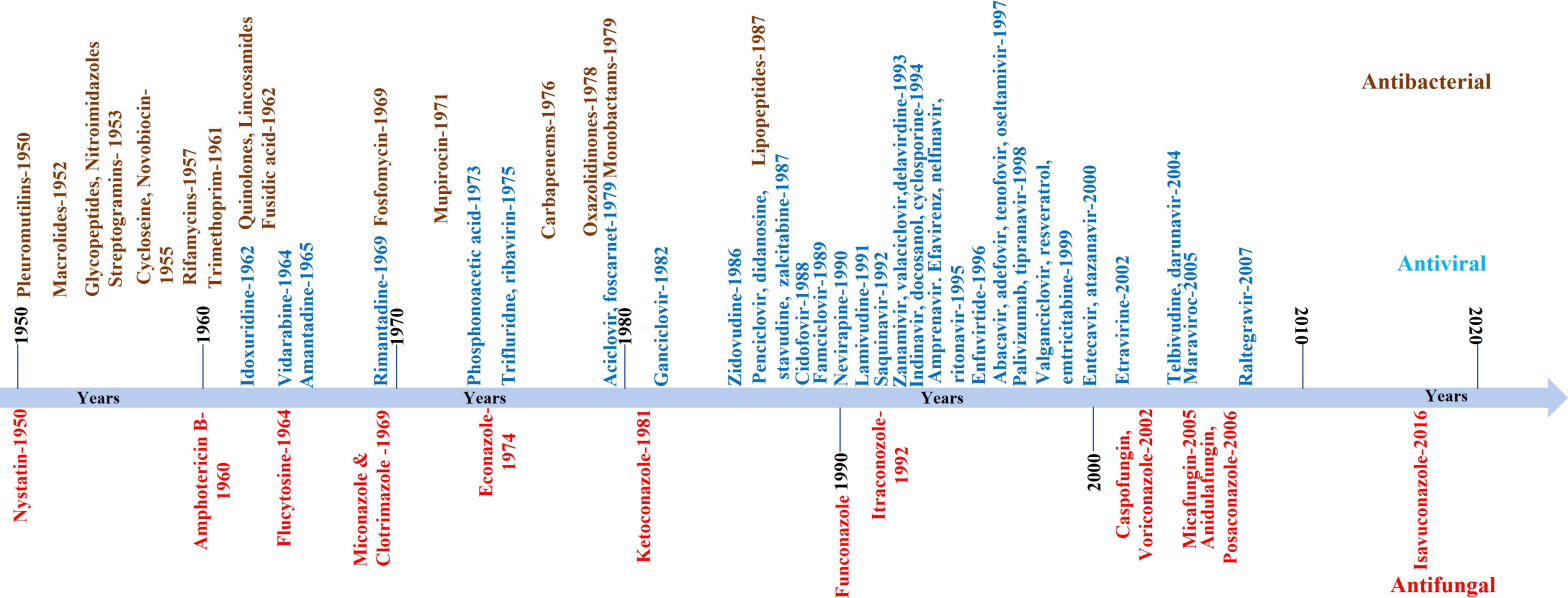
Schematic showing the pace with which anti-fungal compounds were developed or identified. In the Figure, we only listed those compounds approved for clinical use. The compiled list is from 1950 onwards. For comparison, antiviral and antibacterial compounds are also shown.

The continuous increase in global fungal infection or burden is one problem. The availability of a limited number of clinically approved anti-fungal drugs and the persistent rise in the resistance against these drugs is another big problem for clinicians and pharma companies (Wiederhold, 2017; Fisher et al., 2022; Mcdermott, 2022). For example, the first anti-fungal molecule was identified in the 1950s, and till today less than 20 anti-fungal molecules are licensed for use in clinics. Contrary to this, more antiviral or antibacterial molecules are available. A comparative pace with which anti-fungal molecules are identified compared to antibacterial or antiviral is shown in **Figure 2**. Apart from approved anti-fungal drugs (**Figure 2**), several candidate molecules are being evaluated in different stages of drug development, discussed elsewhere (Bouz and Doležal, 2021). We have not discussed those molecules in the present review as they are not approved for clinical use.

Unlike bacteria and viruses, targeting fungi with chemical drugs is difficult. This problem stems from the fact that both fungus and host (in this case, humans) cells are eukaryotic, thus making discovering or developing new anti-fungal molecules safe for human use is slow and laborious (Ostrosky-Zeichner et al., 2010; Roemer and Krysan, 2014; Seneviratne and Rosa, 2016). The eukaryotic nature of fungal cells means essential cellular and biochemical pathways (including key proteins) are conserved (Candau et al., 1996; Kachroo et al., 2015; Kumar, 2018a). Sometimes proteins may not be conserved, but conserving small motifs or folds is also possible (Thaller et al., 1998; Sousounis et al., 2012). These conserved motifs in non-homologous proteins become the reason for drug toxicities. Besides scientific issues hampering the identification or discovery of novel molecules with anti-fungal activity, challenges associated with designing and evaluating clinical trials remain a big hurdle (Rex et al., 2001). Unfortunately, these fundamental challenges are in addition to the well-documented scientific, economic, and regulatory challenges that face the development of anti-infectives in the general (Boucher et al., 2009).

Ideal targets for drug discovery are those present in pathogens while their complete absence from host cells. The same principle is used to identifies or develops molecules with an anti-fungal activity (Odds et al., 2003). Unfortunately, the eukaryotic nature of fungal cells left us with very few pathways and proteins that can act as a target for anti-fungal drug discovery (Perfect, 2017). One of the molecular pathways commonly targeted is fungal lipid biosynthesis. Unlike human cells, whose membranes have cholesterol, fungal cells possess ergosterol. Therefore, enzymes involved in ergosterol biosynthesis are used as a target for medical intervention. Apart from lipids, fungal cells include cell walls of chitin, glucans, mannans, and glycoproteins, which are absent in human cells. Therefore, pathways and enzymes involved in fungal cell wall synthesis are important molecular targets for screening anti-fungal molecules. Besides these common pathways, newer pathways and targets have been identified. For example, the calcineurin pathway (Juvvadi et al., 2017), the sphingolipid synthesis pathway (Hast et al., 2011), th**e** RAS pathway (Mor et al., 2015), trehalose synthesis pathway and others as discussed elsewhere (Perfect et al., 2017).

The availability of high-quality complete genome sequences and parallel advances in bioinformatic tools allowed one to look for the ORF present only in the fungal genome while absent from the host genome. These fungal-specific ORF may also act as a target for the discovery of novel anti-fungal molecules (Hopkins and Groom, 2002; Abadio et al., 2011). This can be a new direction in developing novel anti-fungal molecules or compounds. This may also help better understand fungal biology and the possible role of those ORF. This approach may increase the molecular targets available for discovering anti-fungal drugs. Hence genome mining of fungal species and identifying fungal-specific ORF is gaining significant importance as a starting point for drug discovery against fungal infection.

## 5 Fungal treatment strategies

**Figure 3:**
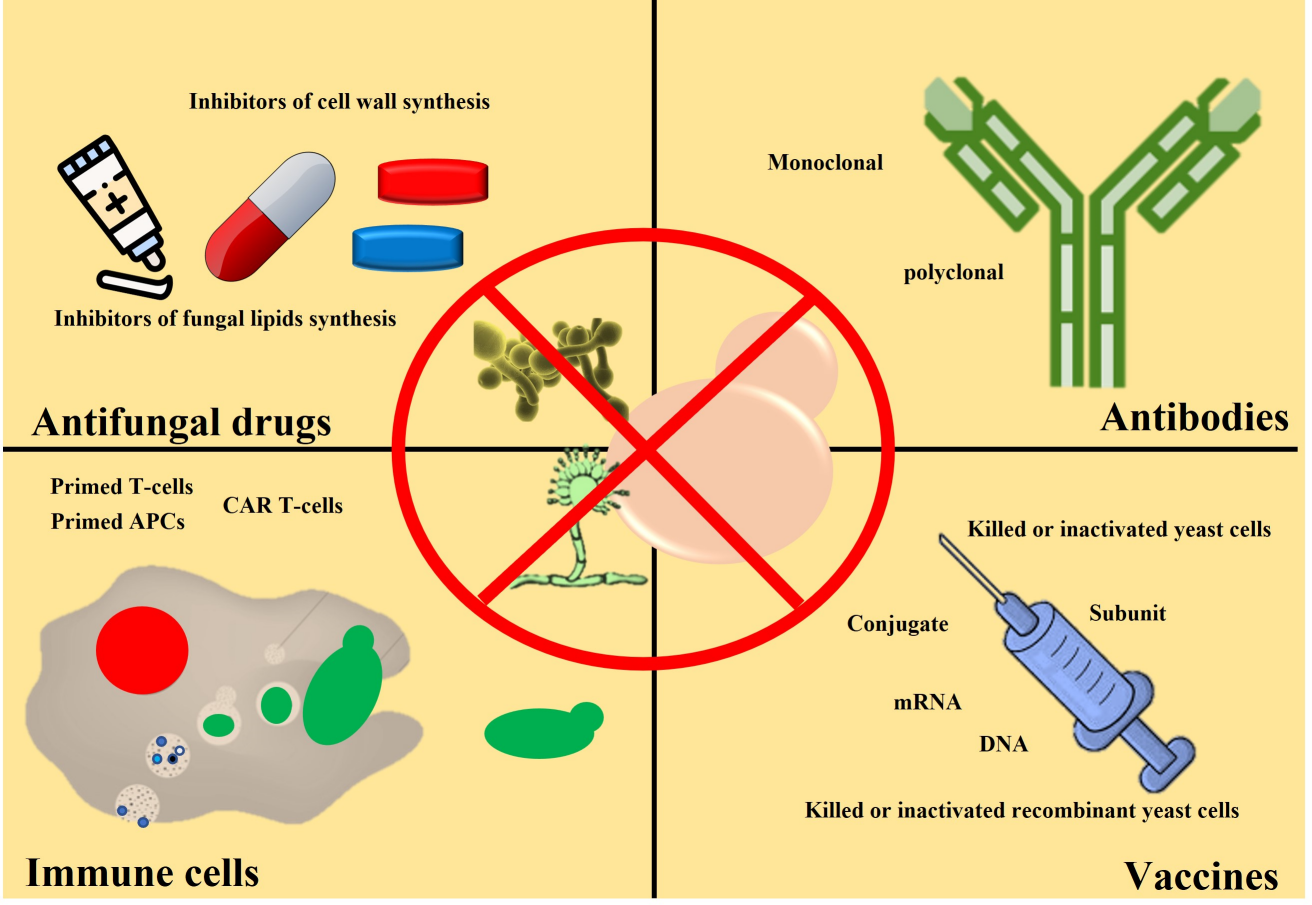
Schematic showing different clinical approaches to reduce fungal burden. Anti-fungal drugs (capsules or pills) are commonly used to treat fungal infections. Anti-fungal cream is also used (for example, skin burn or damage). So far, no anti-fungal vaccine is available for clinical use, but it may become available. Antibodies and activated immune cells are under investigation stage only.

In the previous section, we mentioned the global fungal burden and its impact on human health and society. We also mention the reasons for the increasing fungal burden and a possible rise in anti-fungal resistance. Different approaches have been taken to address the issue of the bust in global fungal burden and rise in anti-fungal resistance, including identifying novel molecules with anti-fungal activity, using antibodies, active immune cells, and vaccines. We will discuss both the advantages and limitations of each approach. However, the discussion on fungal infection and associated immune response will be skipped as this area falls outside the scope of this review and has been discussed previously (Nami et al., 2019). Herein we highlight commonly used methods to treat fungal infections. Different strategies used to fight against fungal infection and ways to reduce fungal burden are shown in **Figure 3**.

### 5.1 Anti-fungal molecules

Using small molecules with anti-fungal activities is the most common and oldest way of treating fungal infections. In all the cases, molecules that inhibit or hamper fungal growth by targeting pathways specific to fungal cells but absent in human cells are used. For example, most anti-fungal drugs approved for use in humans target either fungal lipid biosynthesis pathways (Amphotericin B) or chitin-based fungal cell wall synthesis pathways (for example, Echinocandins). Although few anti-fungal also perturb other fungal cell processes like mitosis (for instance, Griseofulvin), nucleotide synthesis inhibitors (for example, Flucytosine), and protein translational (for example, Tavaborole). The detailed discussion about their structure and mode of action is beyond the scope of this review and can be found elsewhere (Mazu et al., 2016; Scorzoni et al., 2017). Apart from these pathways or molecular targets, efforts were made to identify newer targets for anti-fungal drug discovery (Mccarthy et al., 2017). Besides screening available chemical libraries, screening natural products to identify biomolecules with anti-fungal activity is gaining significant importance. Biomolecules with anti-fungal properties originating from living systems, including bacteria, algae, plants, sponges, and even fungi, are now reported more frequently (Aldholmi et al., 2019).

Although small molecules with anti-fungal activity remain the first and most important line for treating fungal infection, the continuous and rapid development of resistance against these molecules remains a significant problem (Nucci and Perfect, 2008; Fisher et al., 2022). Further, many anti-fungal drugs may have toxicity in humans, especially those that target pathways other than fungal lipid or cell wall synthesis (Lewis, 2011; Tverdek et al., 2016). To address the issue of anti-fungal resistance, newer small molecules with anti-fungal activity targeting either the same pathways or novel protein complexes are performed on an extensive scale. An approach involving modifying already available anti-fungal molecules and repurposing drugs is also under investigation. Even the combination of two or more anti-fungal medications to treat the patient when a single medicine fails to give a satisfactory outcome is also getting significant acceptance in clinics (Johnson and Perfect, 2010; Vandeputte et al., 2012; Kim et al., 2020; Fisher et al., 2022; Mcdermott, 2022). Furthermore, to minimize the use and dependency on small molecules and to tackle the issue of anti-fungal resistance, immuno-based methods (including the use of antibodies and activated immune cells) and the application of anti-fungal vaccines is also proposed (see following sections) (Fisher et al., 2022).

### 5.2 Use of antibodies

As mentioned above, to minimize the dependency on small molecules and better address the problem of anti-fungal resistance, immunological methods involving antibodies and activated immune cells (discussed in the following sub-section) are now under investigation. In the antibodies-based approach to treating fungal infection, monoclonal antibodies are infused into the infected individual (Larsen et al., 2005). Since this approach involves clinical procedures, this can only be adopted on a small scale. It is costly and requires well-trained medical staff and facilities. Before this procedure, proper antibody potency or efficacy must be tested thoroughly. A slight change in antibody may affect its efficacy (Bugli et al., 2013). However, this approach can be helpful if all the available anti-fungal drug fails to give satisfactory outcomes. The application of antibody therapy showed significant improvement even in HIV-positive individuals (Larsen et al., 2005). Combining the anti-fungal drug with antibody therapy may be an excellent choice for the most satisfactory results. To get even better protection, it is recommended to use a cocktail of different monoclonal antibodies or a population of different polyclonal antibodies. A single monoclonal antibody may easily miss the target in case of even changes of a few amino acid residues in the epitope. Collecting antibodies from the patient who recovered from fungal infection and transfused into fungal patients under treatment will also be interesting. However, before this, performing the required hematological tests is crucial. However, applying antibodies (passive immunization) does not provide long-term protection and can be used only for curative purposes.

### 5.3 Use of immune cells

In cell-based therapy, immune cells (like dendritic cells) are first primed or activated *ex vivo* and injected into the individual to protect against fungal infection (Bozza et al., 2004; Perruccio et al., 2004). Example, in study involving immune cells, it was observed that the adoptive transfer of T-cells protects against *Candida*, *Aspergillus*, and *Mucormycetes* in pre-clinical animal studies (Tramsen et al., 2013). Like antibodies-based therapy, this approach also needs well-trained medical personnel and facilities. It is also required the drawing of blood and its transfusion into patients. It may also involve separating required immune cells and their injection into a patient. Again, it can be adopted for a limited number of people. Also, prior checking of blood for any infection and other hematological issues is required when activated immune cells are taken from recovered patients. Additionally, blood type matching may also require, which is also one of the limitations of this approach. Again, this is curative and do not provide long-term protection.

Apart from primed immune cells, several studies showed the possible use of CAR-T cell therapy to treat invasive fungal infections (Kumaresan et al., 2014). Detailed discussion on this technology is beyond the scope of the present review and can be found elsewhere (Garner et al., 2021). This strategy for fungal infection is costly (as of now), requires sophisticated technology, high-end facility, and personnel, and cannot be adopted on a mass scale.

## 6 Anti-fungal vaccines

So far, small molecules with anti-fungal activity remain the only way to fight and treat fungal infections. Besides small molecules with anti-fungal activity, immunological methods (antibody and cell therapy discussed above) are also being tested to fight against fungal infection. However, these immunological therapies are of limited scope. Therefore, there is an urgent need for alternative strategies to treat or prevent fungal infections. Looking at all possible ways to control or prevent microbial infection, mass immunization with vaccines remains an ideal way. Unfortunately, unlike the availability of vaccines against common viral or bacterial infections, there is no approved vaccine against any fungal infection (Perfect, 2017). This raises several questions. Is it possible to develop a vaccine against fungus? Were efforts made to develop anti-fungal vaccines? In the past, efforts were made to develop anti-fungal vaccines, which may soon become a reality. This section will discuss the different approaches used to develop anti-fungal vaccines. We will also discuss the advantages and disadvantages of each platform and update readers about the present status of potential anti-fungal vaccines, which are in different stages of development.

The platforms used to develop anti-fungal vaccines range from traditional (like attenuated yeast cells, use of dead or killed cells) to modern-day platforms (like subunit, recombinant, conjugated, and nucleic acid-based vaccines). A list of previous studies involving the development of anti-fungal vaccines is given in **Table 1**. The advantages and disadvantages of each vaccine development platform were discussed previously (Kumar and Kumar, 2019). Here we will discuss the studies which showed the possibility of developing anti-fungal vaccines.

**Table 1:** List of studies associated with the development of anti-fungal vaccines.

Fungal disease	Antigen	Type of vaccine	Status	Reference
Candidiasis	Als3p Als1p	Recombinant	Phase I	(Pappagianis, 1993; Spellberg et al., 2006; Lin et al., 2009; Baquir et al., 2010; Schmidt et al., 2012; Ibrahim et al., 2013; Singh et al., 2019)
	SAP2	Recombinant	Pre-clinical	(Sandini et al., 2011; De Bernardis et al., 2012)
	Secreted aspartyl proteinase protein, Sap2p PEV-7	Recombinant	Pre-clinical	(Sandini et al., 2011)
	Tet-NRG1 (*C. albicans* strain)	Genetically engineered/Live attenuated	Pre-clinical	(Martínez-López et al., 2008; Saville et al., 2009)
	*C. albicans* PCA-2 strain	Live-attenuated	Pre-clinical	(Bistoni et al., 1986)
	Cell wall surface proteins (CWSP)	Subunit	Pre-clinical	(Carneiro et al., 2016)
	*C. albicans* Mannan extracts	Conjugate	Pre-clinical	(Han et al., 1999)
	Laminarin (Lam) β-glucan	Conjugate	Pre-clinical	(Han et al., 1999; Chiani et al., 2009; Torosantucci et al., 2009; Bromuro et al., 2010; Pietrella et al., 2010)
	B-1,2 mannan and (rAls1p-N)	Conjugate	Pre-clinical	(Liao et al., 2019)
	*C. dubliniensis* mannan/Human serum albumin (HSA)	Conjugate	Pre-clinical	(Paulovičová et al., 2007)
	Fructose bisphosphate aldolase (Fba) (cytosolic and cell wall peptides)	Subunit	Pre-clinical	(Cutler et al., 2011) (Xin and Cutler, 2011)
	*C. albicans* serotypes a and b ribosomes	Recombinant/Conjugate capsule	phase II	(Levy et al., 1989)
	Heat-killed *C. albicans* (HK-CA)	Subunit/ Conjugate	Pre-clinical	(Cárdenas-Freytag et al., 1999)
	Glycolytic enzyme enolase	Recombinant	Pre-clinical	(Qing Li et al., 2011)
	65 kDa mannoprotein (Camp65p)	Subunit/ Conjugate	Pre-clinical	(Sandini et al., 2007)
	*C. albicans* cell surface protein Hyr1	Recombinant	Pre-clinical	(Luo et al., 2011)
	*C. glabrata* secreted proteins	Secreted proteins	Pre-clinical	(Kamli et al., 2022)
	*C. albicans* cells	Heat-inactivated whole cells	Pre-clinical	(Martin-Cruz et al., 2021)
	Combining β-mannan and peptide epitopes	Subunit/ Conjugate	Pre-clinical	(Xin et al., 2008)
Aspergillosis	*Aspergillus fumigatus* crude culture filtrate Ags	Sonicate and filtrate Ags	Pre-clinical	(Cenci et al., 2000)
	Asp f3	Subunit/recombinant	Pre-clinical	(Diaz-Arevalo et al., 2011)
	*Aspergillus fumigatus* viable conidia	Sonicate and filtrate Ags	Pre-clinical	(Ito and Lyons, 2002)
	*Aspergillus fumigatus* hyphal sonicate (HS)	Recombinan	Pre-clinical	(Ito et al., 2006)
	Heat killed yeast (HKY) of *S. cerevisiae*	Live-attenuated	Pre-clinical	(Clemons et al., 2014a; Clemons et al., 2014b)
	*A. fumigatus* epitope p41 from the cell wall glucanase (Crf1)	Subunit	Pre-clinical	(Stuehler et al., 2011)
	Asp 16 f	Recombinant/Subunit	Pre-clinical	(Bozza et al., 2002)
	Asp 3 f	Recombinant/Subunit	Pre-clinical	(Ito et al., 2006)
	Proteins: Gel1p, Crf1p, Pep1p, Cat1p, Sod1p, Dpp5p, RNUp, Mep1p, Polysaccharides: _1–3 glucan, _1–3 glucan, GM, Glycolipids: GSL, LGM	Recombinant/Subunit	Pre-clinical	(Bozza et al., 2009)
Pan fungal	β-glucans of *S. cerevisiae*	Heat Killed Yeast (HKY)	Pre-clinical	(Liu et al., 2011)
Blastomycosis	Adhesin BAD1 gene	Whole organism/Live-attenuated	Pre-clinical	(Wüthrich et al., 2003)
Paracoccidioidomycosis (PCM)	(PCM) gp 43 (P10)	DNA vaccine (pcDNA3-P10)	Pre-clinical	(Pinto et al., 2000; De Amorim et al., 2013)
	(PCM) gp 43 (P10)	Recombinant protein	Pre-clinical	(Assis-Marques et al., 2015)
	P10- FliC fusion protein	Recombinant	Pre-clinical	(Braga et al., 2009)
	rPb27	Recombinant	Pre-clinical	(Fernandes et al., 2011)
	Heat shock protein 60 (HSP60)	Recombinant	Pre-clinical	(De Bastos Ascenço Soares et al., 2008)
	*Mycobacterium leprae* derived HSP65	Recombinant DNA	Pre-clinical	(Ribeiro et al., 2010)
Cryptococcosis	GXM	Conjugate/Soluble antigenic fractions	Pre-clinical	(Devi, 1996)
	GalXM	Subunit/Conjugate	Pre-clinical	(Chow and Casadevall, 2011)
	*C. neoformans* strain H99γ (serotype A, Matα)	Live-attenuated	Pre-clinical	(Wozniak et al., 2011)
	Mutant *C. neoformans* strain lacking the enzyme sterylglucosidase 1 named (Δsgl1)	Live- attenuated- recombinant	Pre-clinical	(Rella et al., 2015)
	CneF (culture filtrate Ags), Mannoprotein	Subunit/Recombinant	Pre-clinical	(Specht et al., 2007)
	GXM	GXM–protein conjugate	Pre-clinical	(Oscarson et al., 2005)
	P13 (a peptide mimetic of GXM)	Conjugated	Pre-clinical	(Zhang et al., 1997; Fleuridor et al., 2001; Pirofski, 2001; Beenhouwer et al., 2002; Maitta et al., 2004; Datta et al., 2008)
	*Cryptococcus neoformans* fbp1Δ	Heat-Killed cells	Pre-clinical	(Wang et al., 2019)
	Laminaran	Subunit (algal β glucan based)	Pre-clinical	(Rachini et al., 2007)
Pneumocystis pneumonia	Kexin genes	Kexin-CD40 L DNA vaccine	Pre-clinical	(Zheng et al., 2005)
	P55 protein (major surface glycoprotein)	Recombinant	Pre-clinical	(Feng et al., 2011)
	Major surface glycoprotein (also known as gp120)	Recombinant	Pre-clinical	(Theus et al., 1998)
Histoplasmosis	Water-soluble ethylenediamine extract from cell wall	Inactivatedfiltrated Ags/Soluble antigenic fractions	Pre-clinical	(Garcia and Howard, 1971)
	Ribosomes or live yeast cells of *H. capsulatum*	Live-attenuated	Pre-clinical	(Tewari et al., 1977)
	Cell wall and cell membrane of yeast-phase *H. capsulatum* G217B	Live-attenuated	Pre-clinical	(Gomez et al., 1991)
	Histone H2B–like protein	Live-attenuated/Recombinant	Pre-clinical	(Nosanchuk et al., 2003)
	Heat Shock Protein 60 (HSP-60)	Recombinant	Pre-clinical	(Deepe et al., 2002; Scheckelhoff and Deepe, 2002)
	HIS-62	Recombinant	Pre-clinical	(Gomez et al., 1991)
	80-kilodalton antigen	Recombinant	Pre-clinical	(Gomez et al., 1992)
	Sec31 homologue	Recombinant	Pre-clinical	(Scheckelhoff and Deepe, 2006)
	H antigen (*H. capsulatom*)	Recombinant antigen	Pre-clinical	(Deepe and Gibbons, 2001)
Coccidioidomycosis	Killed spheroles	Whole organism/Inactivated	Phase III	(Pappagianis, 1993)
	*C. immitis* spherule-phase genes	DNA	Pre-clinical	(Ivey et al., 2003) (Zheng et al., 2005)
	T-cell epitopes Antigen 2/proline rich Ag (Ag2/PRA)/Chimeric polyprotein	Recombinant protein	Pre-clinical	(Abuodeh et al., 1999) (Shubitz et al., 2006) (Tarcha et al., 2006)
	Attenuated mutant (ΔT vaccine strain)	Live-attenuated	Pre-clinical	(Hung et al., 2011)
	Immunodominant T cell epitopes	Recombinant	Pre-clinical	(Hurtgen et al., 2012)
	*C. posadasii* Gel-1 (β 1,3 glucosyltransferase)	Recombinant protein	Pre-clinical	(Delgado et al., 2003)
	Urease (rURE)	Recombinant	Pre-clinical	(Li et al., 2001)
	Spherule phase of *C. posadasii* Peroxisomal matrix protein (Pmp1)	Recombinant	Pre-clinical	(Orsborn et al., 2006)
	Chimeric protein-aspartyl proteinase, phospholipase B and α mannosidase	Recombinant protein	Pre-clinical	(Tarcha et al., 2006)

### 6.1 Live attenuated or killed whole yeast-based vaccines

Unlike some of the species of bacteria (for example, *Mycobacterium leprae*, *Mycobacterium tuberculosis*) and protozoa (for example, species of *Plasmodium*) which defies conventional or available methods of culture on a commercial scale, species of common pathogenic fungi can be grown on a large scale (example species of *Candida*). This allowed the development of a whole yeast-based vaccine. Several studies in the past already showed proof of concept. For instance, it was observed that the administration of heat-killed yeast (HKY) provides effective protection against aspergillosis and coccidioidomycosis (Capilla et al., 2009; Stevens et al., 2011). However, several pathogenic yeasts remain challenging to grow on a large scale and therefore need different approaches to target such yeast species (see the following sections). Further, other platforms were explored to prevent the need for growing a pathogenic entity on a large scale.

### 6.2 Recombinant yeast

Usually, regular harmless yeast is used as a host to express proteins from pathogenic fungi. The recombinant yeast was then injected, and efficacy was noted. For example, the injection of budding yeast expressing *P. brasiliensis* gp43 was able to protect mice from paracoccidioidomycosis (PCM) (Assis-Marques et al., 2015). A vaccine preparation using hemolysin expressed in *the S. cerevisiae* vector has been reported to confer partial protection in an infection model of coccidioidomycosis (Capilla et al., 2009). One can use cells expressing heterologous protein intracellularly or as yeast display or both approach combines. The advantage of this approach is that the basic nature of the fungal cell wall is conserved across fungal species; therefore, even the budding yeast cell will act as a suitable adjuvant. Thus, preventing the need for adding adjuvants makes vaccine development more straightforward and economical. Note that the level of immune response is independent of yeast’s live or dead nature. Still, due to the safety point, yeast cells are heat-inactivated before administration into animal or human subjects. Since heat inactivation is easy, simple, and economical, it prevents using chemicals like formalin, commonly used to attenuate the pathogen during vaccine development (Kumar, 2018b; Kumar and Kharbikar, 2021).

On top of that, several studies already showed the integrity and stability of heterologous proteins and native yeast proteins during heat inactivation (Kumar and Kharbikar, 2021). The long-term stability of heterologous proteins in yeast cells, even at ambient temperature, allowed the use of yeast cells as a biodegradable immunogenic microcontainer (Kumar et al., 2022a). Heterologous expression of proteins from different pathogenic fungi in budding yeast and the use of whole recombinant yeast can be an excellent way to mount immunity against fungal infection.

### 6.3 Subunit vaccines

Subunit vaccines use only one or a few components of the pathogen. It can be DNA, mRNA, protein, peptide, or glycan from the pathogen. Since this approach uses only a few components from the pathogen, it is relatively safer and does not require pathogenic entity handling. However, this may require cloning, expressing, and purifying the protein. Subunit vaccine formulation needs the addition of stabilizer, adjuvant, and other components, including preservatives. Because of their non-particulate nature, subunit vaccines showed rapid or fast body clearance, and immune cells are not good at the uptake of soluble immunogens. Therefore, subunits vaccines may sometimes fail to mount a robust immune response and thus need modification (see the following subheading). However, despite all these issues, several studies showed encouraging results and provided protection against Candidiasis (Cutler et al., 2011; Xin and Cutler, 2011), Aspergillosis (Stuehler et al., 2011), Cryptococcosis (Specht et al., 2007). A detailed discussion of peptide-based vaccines for fungal diseases can be found elsewhere (BR Da Silva et al., 2020).

### 6.4 Conjugate vaccines

Conjugate vaccines are also subunit vaccines involving only a few components from pathogens. In conjugate vaccines, a weak immunogen is attached covalently to a stronger immunogen acting as a carrier. The immunogen from the pathogen provides specificity and carrier help in mounting a robust immune response. Therefore, conjugate vaccines can be considered the next level in subunit vaccines. Using this approach, several studies showed promising results and were able to protect against Candidiasis (Chiani et al., 2009; Torosantucci et al., 2009; Bromuro et al., 2010; Pietrella et al., 2010) and Cryptococcosis (Beenhouwer et al., 2002; Datta et al., 2008).

### 6.5 Nanotechnology

Nanotechnology is a relatively new approach to vaccine development. An immunogen of interest is bound or coated onto nanomaterial (nanoparticles or beads). Because of its particulate nature, this approach is better in raising immune response than soluble protein immunogen. For example, applying plasma bead coated with *C. albicans* cytoplasmic proteins provides a positive immune reaction against *Candida* infection (Ahmad et al., 2012). Again, this may require expressing and purifying the immunogen of interest followed by coating it onto the nanoparticle. The amount of immunogen that can be loaded on a nanoparticle and whether a given nanoparticle can be used for different immunogens need further investigation. The detailed discussion on the application of nanotechnology for developing anti-fungal vaccines is beyond the scope of the present review and can be found elsewhere (Kischkel et al., 2020).

### 6.6 Nucleic acid-based vaccines

Nucleic acid-based vaccines are the newest entry in the vaccine formulation strategy. Nucleic acid-based vaccines can be DNA-based, or mRNA based, depending on the nucleic acid used in vaccine formulation. The best example of mRNA-based vaccines is those used against COVID-19 (manufactured by Pfizer and Moderna). So far, no DNA-based vaccine has been approved for clinical use. In the future, we may see the use of DNA-based vaccines in clinical settings as several pre-clinical studies showed encouraging results when targeted against fungal infection (Ivey et al., 2003; Zheng et al., 2005).

### 6.7 Pan fungal vaccine

The conserved nature of cell wall components, particularly polysaccharides like beta-glucan, mannans, and zymogen, and their absence in the host, including mammals, and poultry birds, make them suitable for inducing the immune response against fungal infection. Apart from the conserved nature of the fungal cell wall and many associated proteins, components of the cell wall are suitable immunogens and make the cell wall a promising adjuvant. These features suggest that even a common budding yeast with a similar cell wall should be able to mount an immune response and should it protect from other fungal species. Thus, a given anti-fungal vaccine (with inactivated yeast) can be used against several fungal infections. Several studies validate this assumption that heat-inactivated budding yeast could mount a robust immune response and provide protection against other fungal infections (Liu et al., 2011). In another strategy to develop an anti-fungal vaccine with broad application, an antigenic component of the fungal cell wall is conjugated with a carrier protein. For example, one study showed that the covalent attachment of fungal cell wall β-glucans to diphtheria toxin could mount a robust immune response and provide protection against two common fungal pathogens (Torosantucci et al., 2005). Thus, it may be possible that only a few anti-fungal vaccines may protect against almost all common pathogenic fungi.

## 7 mRNA-based anti-fungal vaccines

Despite the dramatic success of mRNA-based vaccines in preventing the spread of SARS-CoV-2 infection during the COVID-19 pandemic, it is surprising that there is no study where this platform has been tested to fight against fungal infections (Bruch et al., 2022). However, several studies are available where DNA-based vaccines were used to prevent fungal infection (**Table 1**). Like a recombinant, conjugate, and subunit-based anti-fungal vaccine, we may soon come across studies where mRNA-based technology will be used to develop anti-fungal vaccines. Indeed, using mRNA-based technology to develop an anti-fungal vaccine is complex and needs more work in identifying suitable protein immunogen(s) and safe adjuvant. Regarding an adjuvant, in the case of anti-fungal vaccine(s), the fungal cell wall components can be used as an adjuvant. The protein immunogen used as a vaccine should be highly immunogenic and not share any similarity with host proteins, ideally conserved in broad fungal species. One may go with a panel of proteins (and not just one immunogenic protein) if needed.

**Figure 4:**
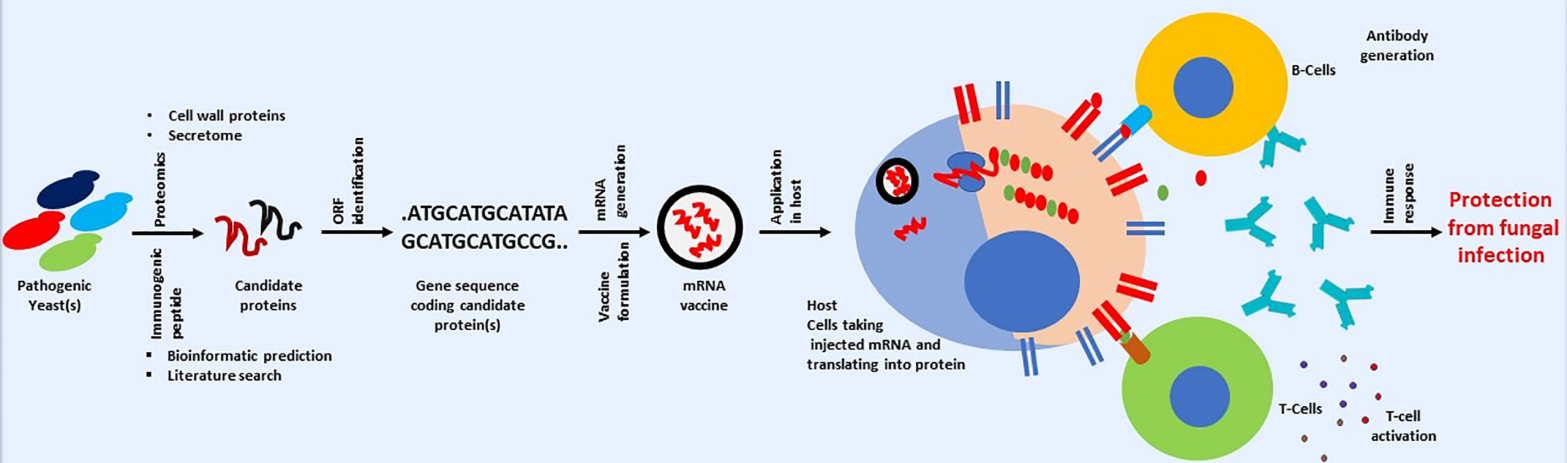
Cartoon presentation showing a possible workflow for developing a mRNA-based anti-fungal vaccine. Schematic is only for conveying the primary message. Some steps involved in mRNA-based vaccine development against fungal infection may vary.

Therefore, finding or identifying fungal proteins that fulfill the above criteria can be a significant roadblock in developing an anti-fungal vaccine using mRNA-based technology. However, the combined application of genomics and proteomics can be helpful in finding a candidate’s immunogen protein (Thomas et al., 2006). Fungal proteomics can be used to identify surface or cell wall proteins (Asif et al., 2006). Identified proteins can be checked for immunogenicity using bioinformatics or other available *in silico* predicting tools (Poran et al., 2020). The same bioinformatics tools can be used to check the similarity of candidate proteins. Those that show any similarity (with host protein) should be dropped, and the remaining should be taken further. The gene encoding for the remaining protein should be searched in the fungal genome. The availability of nucleotide sequences will help downstream processes, including mRNA synthesis (Pardi et al., 2018). A broad pipeline for developing mRNA-based anti-fungal vaccines is shown in **Figure 4**. One can look for previously published fungal cell wall proteomic studies or fungal secretomes (Choi et al., 2010; Rasheed et al., 2019; Gong et al., 2023).

Apart from looking at previously published fungal cell wall/surface or secretome proteomics, one can also take fungal protein used in previous studies related to developing anti-fungal vaccines with positive or encouraging outcomes in pre-clinical or clinical studies (**Table 1**). For example, genes used in the last DNA-based vaccine development can be a good starting point in developing a mRNA-based anti-fungal vaccine. The DNA sequence in previous studies can be used for mRNA synthesis, which can be used in developing anti-fungal vaccine. This is important as information about immunogenicity in human subjects or animal models, efficacy, and safety is already available. Therefore, one can take any path in checking the possibility of developing mRNA-based anti-fungal vaccines. Although vaccines offer several advantages in fight against infectious diseases, but it is important to mention that vaccines also suffer from several issues like thermolabile nature, need for cold chain for storage and distribution and so on. Issues associated with vaccines are discussed separately (Kumar et al., 2022a; Kumar et al., 2022b). It is surprising to note that study related to use of virus like particle for developing anti-fungal vaccines is lacking, although this approach is successfully applied against several infectious diseases (reviewed by Srivastava et al., 2023).

## 8 Conclusion

From all the information mentioned above, it can be very well justified that fungal infections are as common as bacterial or viral infections. However, unlike bacterial or viral infections, fungal infections are not documented or appropriately addressed. If recorded or reported correctly, it may be possible that deaths associated with all fungal infections may surpass the ones related to common infectious diseases due to bacteria (for example, *Mycobacterium* sp.), protozoa (for example, *Plasmodium* sp.) or viral infections which gets much more attention and have a proper policy for their monitoring (Brown et al., 2012). Treating fungal infections is becoming more problematic due to the persistent rise in resistance against limited anti-fungal drugs. Since several factors increase the global fungal burden, we need to adopt or formulate a policy (at both international and local levels) addressing all the elements contributing to the rise in global fungal burden to avoid any fungal endemic or possibly pandemic.

The availability of refined genome sequences of both host (human) and pathogenic fungal species makes genome mining possible, thereby identifying ORFs unique and specific to only the fungal domain. Those ORF need to be studied and used as a target in discovering anti-fungal drugs where possible. For example, several data mining studies have shown the presence of ORF unique to only *S. cerevisiae* or budding yeast and their absence from the mammalian genome (Hopkins and Groom, 2002). This may speed up the discovery of new anti-fungal molecules. Combining genome mining and better prediction of structures (including motifs and folds) may also help develop an anti-fungal drug with fewer side effects or toxicities, as candidate target proteins sharing motifs or folds with host proteins can be dropout at early stages of drug discovery. Said this, identifying anti-fungal molecules remains expensive and lengthy, and therefore world needs a safe and effective alternative for treating or preventing fungal infection.

Owing to the success of vaccines against viral and bacterial infection, it is worth developing anti-fungal vaccines. Anti-fungal vaccines, as a preventative measure, will reduce society’s economic burden and fungal load and help better manage anti-fungal resistance. The vaccine will also help save resources, time, and efforts needed to develop or identify anti-fungal molecules as modern vaccine development technology is safer and more rapid, as seen in the case of mRNA-based vaccines against COVID-19. Although many studies showed the utility of antibodies and cell therapy for fungal infections, the cost and scale to which these immunotherapies can be done remain an essential question.

A safe and effective anti-fungal vaccine is needed as well as a wish. However, several challenges must be overcome before the safe anti-fungal vaccine is available for public use. Encouraging results from several pre-clinical (or animal studies) and clinical trials of a few potential anti-fungal vaccines suggest that the world may soon have an anti-fungal vaccine approved for public use (**Table 1**). Given the better understanding of host and fungal genomes and parallel advancement in vaccine development platforms, it is no surprise that the world may get its first anti-fungal vaccine at any time soon, maybe in the coming few years. The availability of an anti-fungal vaccine with broad protection (effective against several pathogenic fungi) will be ideal.

## Author contributions

RK conceives the topic idea and initiates writing the draft. VS also contributed to writing. RK and VS prepare the Table and Figures. RK also edited the draft and responded to reviewer’s comments. All authors contributed to the article and approved the submitted version.
